# Effects of Nutritional Education Interventions on Metabolic Risk in Children and Adolescents: A Systematic Review of Controlled Trials

**DOI:** 10.3390/nu12010031

**Published:** 2019-12-21

**Authors:** Rosaura Leis, Carmela de Lamas, María-José de Castro, Rosaura Picáns, Mercedes Gil-Campos, María L. Couce

**Affiliations:** 1 Department of Pediatrics, University Clinical Hospital of Santiago de Compostela, 15704 Santiago de Compostela, Spain; mj.decastrol@gmail.com (M.-J.d.C.); rosaurapicansleis@gmail.com (R.P.); maria.luz.couce.pico@sergas.es (M.L.C.); 2 IDIS-Health Research Institute of Santiago de Compostela, 15704 Santiago de Compostela, Spain; 3 CIBEROBN, Instituto Salud Carlos III, 28029 Madrid, Spain; 4 Facultad de Medicina, Departamento de Pediatría, Universidad de Santiago de Compostela, 15704 Santiago de Compostela, Spain; carmeladelamas@gmail.com; 5 CIBERER, Instituto Salud Carlos III, 28029 Madrid, Spain; 6 Department of Pediatrics, Pediatric Metabolism and Research Unit, Reina Sofia University Hospital, IMIBIC, 14004 Córdoba, Spain

**Keywords:** adolescents, children, dyslipidemia, hyperglycemia, hypertension, insulin resistance, metabolic risk, metabolic syndrome, nutritional intervention, obesity

## Abstract

Childhood obesity is a global public health issue and is linked to metabolic syndrome, which increases the risk of comorbidities such as type 2 diabetes, cardiovascular diseases and cancer. Social, economic and cultural factors influence changes in nutrition and lifestyle characterized by poorer diets and reduced physical activity. This systematic review summarizes the evidence for nutritional education interventions to improve metabolic risks in children and adolescents. Systematic searches of the databases Medline (via PubMed) and Scopus were conducted following PRISMA guidelines. The risk of bias for each study was assessed following the methodology of the Cochrane Collaboration. Ten case-controlled and randomized controlled studies testing nutritional educational interventions targeting children and adolescents from the general population were eligible for inclusion. The sample size was 3915 and the age range was 7–20 years. The duration of intervention ranged from 12 weeks to 20 years. All the studies that provided data on abdominal obesity reported differences in favour of the intervention. However, data on the effects on the remaining components of metabolic syndrome remain inconclusive. These results support the role of nutritional education interventions as a strategy to reduce central adiposity and its possible unhealthy consequences in children and adolescents.

## 1. Introduction

Obesity is a worldwide pandemic that affects all countries, ages and socioeconomic groups. Since 1980, the prevalence of childhood obesity has doubled and tripled in low- and high-income countries, respectively [1,2,3,4]. Closely linked to this widespread health crisis is the increasing prevalence among obese children and adolescents of metabolic syndrome (MetS) [5,6,7,8,9], which includes central adiposity, elevated blood pressure, dyslipidemia and impaired glucose metabolism. MetS is also linked to cardiovascular disease and type-2 diabetes, and MetS in children appears to persist into adulthood [10,11]. Moreover, long term follow-up has shown that 50–80% of obese children become obese adults [12,13], with a high risk of developing the aforementioned morbidities [14,15], in addition to several forms of cancer [16,17]. 

This public health problem is primarily due to changes in nutrition and lifestyle (including poor dietary habits, decreased physical activity, and increased inactivity, primarily linked to screen exposure) influenced by social, economic and physical environments [18,19,20]. The alarm raised by the World Health Organization (WHO) in the 1990s has led to the development of measures to fight the obesity epidemic, including strategies to counteract obesogenic environments [21]. In this regard, the childhood period is a particularly important window of opportunity to establish healthy habits, thereby improving nutritional and metabolic status during the pediatric period and protecting against future chronic diseases. Among the prevention strategies used are education interventions, which can reach large numbers of at-risk children and adolescents during their formative years in a cost-effective manner [22,23]. Importantly, it has been shown that it is more difficult to treat obesity through lifestyle changes in adulthood than in childhood [24].

The relationship between nutritional education interventions in children and adolescents and metabolic risk remains unclear, and data on the clinical outcomes of such interventions are not conclusive. In this systematic review of case-control studies and clinical trials in children and adolescents from the general population, we evaluate the impact of nutritional educational interventions on the main features of metabolic syndrome: abdominal obesity, dyslipidemia, hypertension, fasting hyperglycemia and insulin resistance.

## 2. Materials and Methods 

This review, which is registered in the International Prospective Register of Systematic Reviews (PROSPERO number: ID150393), was designed following PRISMA (Preferred Reporting Items for Systematic Reviews and Meta-Analyses) guidelines [25]. The review question, which was formulated in accordance with the PICOS (Population, Intervention, Comparison, Outcomes, Settings) criteria [26] (Table 1), is as follows: Do nutritional education interventions influence metabolic risk in children and adolescents? 

### 2.1. Literature Search

Articles were selected for inclusion in this review from PUBMED and SCOPUS databases. PUBMED searches were conducted using the following search terms: (“Metabolic Syndrome” (Mesh) OR “Obesity, Abdominal” (Mesh) OR “Dyslipidemias” (Mesh) OR “Hypertension” (Mesh) OR “Hyperglycemia” (Mesh) OR “Insulin Resistance” (Mesh)) AND “Diet, Food, and Nutrition” (Mesh)) AND “Clinical Trial” (Publication Type) AND (“humans” (MeSH Terms) AND (“infant” (MeSH Terms) OR “child” (MeSH Terms) OR “adolescent” (MeSH Terms). For the SCOPUS database, the following search terms were used, excluding results for animal studies: (“Metabolic Syndrome” OR “Abdominal Obesity” OR “Dyslipidemias” OR “Hypertension” OR “Hyperglycemia” OR “Insulin Resistance”) AND “Nutrition” AND “Clinical Trial” AND (“child” OR “adolescent”). 

### 2.2. Inclusion and Exclusion Criteria 

Controlled, randomized or nonrandomized studies of children and adolescents of any ethnicity from the general population published in English or Spanish language between 1 January 1900 and 15 August 2019 were eligible for inclusion. Studies that did not include metabolic risk data, included any additional non-education interventions (e.g., implementation of restrictive diets, changes in school menus, extra physical education classes), were carried out in populations of chronic pathologies including obesity or lacked a control group were excluded from our review. 

### 2.3. Intervention Types

Studies of nutritional education interventions, including those that formed part of broader, multifaceted educational programs implemented in schools, families or communities were selected for inclusion regardless of the duration or intensity of the intervention. 

### 2.4. Primary Outcome Measures

The primary outcome measures for assessing the effects of nutritional education intervention on abdominal obesity were (i) waist circumference (WC) (cm); (ii) changes in waist circumference (cm or %) or waist-to-height ratio; (iii) incidence of abdominal obesity; and (iv) relative risk (RR) of high WC. Effects on plasma lipids were assessed by considering changes in circulating lipids (mmol/L, mg/dL or %) or RR of high triglyceride and low high-density lipoprotein cholesterol (HDL) levels. The parameters considered to evaluate effects on blood pressure were systolic (SBP) and diastolic (DBP) blood pressure (mmHg), percentage changes in SBP and DBP, and changes in the RR of high blood pressure. The effects of interventions on fasting glucose were evaluated based on fasting blood glucose levels or changes in fasting blood glucose (%, mg/dL or mmol/L). Effects on insulin resistance were assessed based on homeostasis model assessment of insulin resistance (HOMA-IR) values, or percentage/absolute changes in HOMA-IR values. 

### 2.5. Study Selection

From the 827 articles obtained from database searches and other sources, 2 authors (M.-J.d.C. and C.d.L.) independently selected studies for inclusion in our review. Discrepancies were arbitrated by R.L., M.L.C., R.P. and M.G.-C. Ultimately, 10 studies [27,28,29,30,31,32,33,34,35,36] were included in the systematic review. 

### 2.6. Data Extraction

From each study the following data were extracted separately by 2 researchers: publication year, number of participants by sex, age, intervention characteristics, trial type and duration, outcome measures, results and conclusions. In cases of a lack of consensus, the remaining authors acted as arbitrators.

### 2.7. Assessment of Risk of Bias 

To assess the risk of bias, 2 authors (M.-J.d.C. and C.d.L.) independently followed the methodology of The Cochrane Collaboration, London, UK [37]. R.L., M.L.C. and M.G.-C. arbitrated in cases of discrepancies of opinion. For each study, the risk of selection bias (random sequence generation, allocation concealment), performance bias (blinding of participants and personnel), detection bias (blinding of outcome assessment), attrition bias (incomplete outcome data), reporting bias (selective reporting) and any other forms of bias was assessed and classified as low, high or uncertain (in cases in which insufficient data were reported). 

## 3. Results

**Figure 1:**
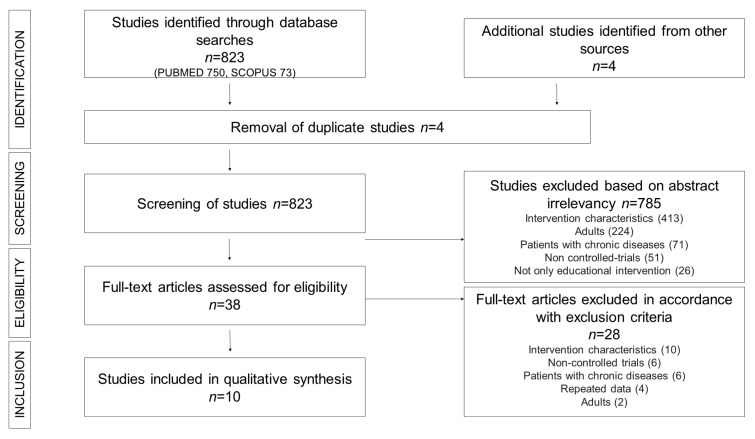
PRISMA Flow diagram of assessment of studies identified in the systematic review.

Figure 1 shows the process applied to select from the 827 initial articles (PUBMED, 750; SCOPUS, 73; other sources, 4) the 10 studies [27,28,29,30,31,32,33,34,35,36] finally included in this systematic review. After deleting four duplicated studies, during the study selection we excluded 785 articles: 413 for not carrying out the educational intervention, 224 because they were performed in adults, 71 for including populations with chronic pathologies, 51 for not being controlled trials and 26 for not only performing educational interventions. Of the 38 full-text articles assessed for eligibility, we excluded 28 (10 for intervention characteristics, six uncontrolled trials, six including patients with chronic diseases, four presented repeated data and two performed in adults). 

### 3.1. Study Characteristics

Table 2, Table 3, Table 4, Table 5 and Table 6 show the main characteristics of the selected clinical trials (two controlled trials [27,33] and eight randomized controlled trials [28,29,30,31,32,34,35,36]), ordered by age and separated, by double line, studies in children and in adolescents. All of the articles were published between 2006 and 2019. The sample size of the study population in the 10 articles was 3915 (range, 37–1733). The age range of the participants was 7–20 years. In one [30] of the articles included in the systematic review, the intervention was performed exclusively on mothers. In six [28,31,33,34,35,36] of the remaining nine studies [27,28,29,31,32,33,34,35,36] in which nutritional education was received directly by children and/or adolescents, families also received some form of nutritional education. Children and/or adolescents received nutritional education within the school program in four [27,28,32,34] of the nine studies [27,28,29,31,32,33,34,35,36] and as an extracurricular activity in the other five studies [29,31,33,35,36]. The duration of intervention ranged from 12 weeks to 20 years. The total time dedicated to the intervention was only indicated in six of the articles, as follows: nine hours [30], 12 h [34], 15 h [27], 18 h [29,33] and 24 h [35]. Two of the studies included in the review [31,36] reported results for the same original data set. Because it could not be guaranteed that some individuals were not included in both subsamples, we included data (abdominal obesity, plasma lipids, blood pressure and fasting blood glucose) from only one of the studies [31] (the study with the highest n value). The follow-up of the intervention was greater than 90% in programs carried out in the schools [27,28,32,34]. Studies carried out as extracurricular activities were generally accepted in the same way [22,33,35,36], except for the large Finnish study that was developed over 20 years [31] and the study carried out in mothers [30]; these two studies accounted a loss greater than 40% of the participants. 

### 3.2. Nutritional Education and Abdominal Obesity

The seven studies [27,28,29,31,32,33,34] that provided data on abdominal obesity (two controlled trials [27,33] and five randomized controlled trials [28,29,31,32,34]) included a total of 3406 children and adolescents (50.4% female) (Table 2). All seven studies reported differences between the control groups and those that received nutritional education; these differences were significant in five studies [27,28,29,32,34]. Four of these five articles [27,29,32,34] reported changes in waist circumference (%, cm or z-score) and the fifth [28] evaluated the incidence (odds ratio) of abdominal obesity. Two of the studies [27,34] also evaluated changes in waist-to-height ratio, but only one reported a significant difference in this parameter [27].

### 3.3. Nutritional Education and Plasma Lipids

Three [29,32,34] of the four randomized controlled trials [29,31,32,34] that reported plasma lipid data found no significant differences between the control and intervention groups (Table 3). One [31] reported a lower RR of high triglyceride levels, but only in boys. Another [34] observed a significantly greater decrease in triglyceride levels in the control group. All four studies, which included a total of 1105 children and adolescents (48.5% females), measured HDL and triglyceride levels. One [29] also measured LDL (low density lipoprotein cholesterol) levels. Three of the studies [29,32,34] compared changes in plasma lipid levels and the fourth [31] compared the RR of high triglyceride levels and of low HDL levels. The three studies that assessed changes in plasma lipid levels reported improvements in HDL levels, although these differences were not statistically significant. 

### 3.4. Nutritional Education and Blood Pressure

Blood pressure data were reported in only three [29,31,33] of the studies included in the review (Table 4), two of which were randomized [29,31]. These three studies included a total of 957 children and adolescents (49.7% female). Each study measured different parameters: changes in SBP and DBP [29]; RR of high blood pressure [31]; and blood pressure after the intervention [33]. Two of the studies reported significant improvements in the intervention groups: one in the RR of high blood pressure [31] and the other in DBP after the intervention [33]. The third study [29], which was the only one in which the intervention was applied exclusively to children without family involvement, reported no significant changes in blood pressure related to the intervention. 

### 3.5. Nutritional Education and Fasting Glucose

Fasting glucose data were reported in six randomized controlled trials [29,30,31,32,34,35], which included a total of 1447 children and adolescents (Table 5). One study [31] assessed the RR of high fasting blood glucose levels, three [29,32,34] evaluated changes in fasting blood glucose, and two [30,35] measured fasting blood glucose levels after the intervention. Only one of the studies [34] observed significant improvements in the group that received nutritional education. The article published by Kong et al. [32] reported an increase in fasting glucose in both the control and intervention groups, but the increase was significantly greater in the intervention group. 

### 3.6. Nutritional Education and Insulin Resistance

Five randomized controlled trials [29,30,32,35,36], including a total of 877 children and adolescents, evaluated the effects of nutritional education on insulin resistance (Table 6). All studies measured HOMA-IR values. Specifically, three studies [29,32,35] measured the mean change in HOMA-IR and two [30,36] measured mean HOMA-IR values after the intervention. Significant improvements were reported in only one study [36]. No significant differences were reported in the three studies [29,32,35] that evaluated mean change in HOMA-IR. 

### 3.7. Risk-of-Bias Assessment

The following articles had the highest risk of biased results: Wadolowska et al., 2019 [27], Davis et al., 2011 [33] and Singhal et al., 2010 [34]. The first two studies [27,33] are non-randomized controlled trials and, therefore, have a high risk of selection bias (risk of bias for random sequence generation and allocation concealment). The third [34] has a high risk of bias due to the lack of allocation concealment: participants were selected after assignment to the control or intervention groups, and the researchers in charge of the selection were not blind to the assignment process. This study also had a high risk of other biases because randomization was performed by clusters (schools), not by individuals. In five of the remaining studies [28,29,30,32,35], we detected a risk of only one type of bias. In the study by Davis et al., 2009 [35], there was a high risk of selection bias due to inadequate random sequence generation: the authors randomized three pairs of twins together in the same group and included data from all three pairs in a total sample of 37 participants. In the study by Costa et al. [30], there was a high risk of attrition bias due to the loss of almost 40% of the study participants. In the other three studies [28,29,32], we detected a high risk of other types of bias: all three studies used cluster randomization rather than individual randomization. For the remaining two studies [31,36], no high risk of any of the biases evaluated was detected. 

The risk of bias of the results of the studies included in this review is, in general, low. Of the 10 studies included, 20% had no high risk of any of the biases assessed [31,36], 50% [28,29,30,32,35] had a high risk of only one type of bias, and the remaining 30% [27,33,34], including the two non-randomized trials [27,33], had a high risk of two of the biases assessed. Further information on the risk-of-bias analyses is provided in the risk-of-bias graphs in the Supplementary Materials (Supplementary Figures S1 and S2).

## 4. Discussion

This systematic review of controlled (CT) and randomized (RCT) clinical trials assesses the effects of nutritional education interventions on the risk of MetS in children and adolescents and reveals a beneficial impact in reducing abdominal obesity. However, data regarding the effects of interventions on the remaining components of MetS, including dyslipidemia, hypertension, fasting hyperglycemia and insulin resistance, are inconclusive. 

The beneficial effect of nutritional education interventions on abdominal obesity in pediatric populations constitutes an important finding for several reasons. First, central adiposity appears to be the predominant diagnostic criteria for MetS [38,39] and related comorbidities [40,41] as reported in the literature. Moreover, abdominal adiposity is an outcome sensitive to changes in nutrition and lifestyle [42,43], which are the main components of the evaluated education interventions. Regarding primary outcome measures, WC is used as an indicator of abdominal adiposity in several published definitions of pediatric MetS (Cook [44], de Ferranti [45], Cruz [46], Ford [47], and International Diabetes Federation Consensus [48]). Only Weiss [49] uses body mass index (BMI) z-scores. In this regard, it should be noted that there are certain drawbacks to using BMI to identify metabolic risk and even obesity. BMI has high specificity but low sensitivity to detect excess adiposity and fails to identify over a quarter of children with an excess body fat percentage [40,50,51]. Assessment of central adiposity by measuring WC, waist-to-height ratio or waist-to-hip ratio (the parameters included in our review) is thus even more important when evaluating a pediatric population [40,41]. Compared with BMI, WC is a better indicator of obesity-related health risk. Thus, for a given WC value, overweight and obese individuals and normal-weight individuals could have comparable health risks [51,52]. It should also be noted that the most recent overview of Cochrane reviews [53] assessing educational interventions in children with overweight and obesity reported a slight reduction in body-weight status, as determined using BMI z-scores, in children of all ages, and states that the decrease in BMI z-score required to ameliorate any comorbidities is unclear, suggesting that the inclusion of body composition indices (e.g., WC) may be useful to this end. Given that current scientific evidence distinguishes between metabolically healthy and unhealthy obese patients [54,55] and normal weight but metabolically obese [56,57,58] individuals, our systematic review has the added value of including an entire pediatric population, not just obese children and adolescents. 

Our abdominal obesity findings indicate that by providing access to a particularly vulnerable age group (children and adolescents from the general population), the school setting offers a valuable opportunity to begin early health promotion and obesity prevention. Some studies have reported that interventions targeting middle or high school pupils are more effective than those focused on elementary schools [34,46,59], while other authors have suggested that interventions targeting children aged 2–5 years may have a greater effect on obesity prevention and management [60]. However, our findings indicate a beneficial impact across all ages evaluated. 

The effects of nutritional education interventions on the remaining components of MetS remain unclear. However, some evidence suggests improved outcomes in lipid profile, specifically in triglycerides, blood pressure, fasting glucose and HOMA IR, more frequently in adolescents. These results may be explained by the fact that nutritional education interventions target changes in diet and lifestyle, which more rapidly modify adiposity, and this is consistent with the beneficial effect observed in abdominal obesity. In addition, alterations in the metabolic profile are slowly and progressively developed over a long time, being more easily detected in pubertal children as reported in the literature [9,61]. Longitudinal studies including longer interventions and follow up times beyond adolescence may help to better track the impact of the nutritional education interventions on these metabolic variables.

Interestingly, of the three included studies that measured blood pressure, the two that reported significant beneficial effects involved interventions targeting both children and their parents. The participation of family members, which may positively condition the health behaviors of children, may explain this beneficial effect. In agreement with these findings, several studies [62,63] have demonstrated that screening for cardiovascular risk (CVR) factors in school children predicts CVR in parents, suggesting not only a genetic component but also a potentially modifiable influence of family lifestyle.

No studies were excluded from our review for a high risk of bias. Although none of the studies indicated whether participants and personnel were blinded, in all cases the outcomes recorded were objective measures, and therefore we concluded that the risk of biased results was low. In several of the included studies, we observed a risk of bias resulting from the randomization process, although all studies sought to minimize this risk (e.g., by selecting similar clusters and/or conducting extensive baseline comparative analyses). 

A key factor to consider is the duration of any intervention and its impact in adulthood. One of the longitudinal studies included in this review conducted the longest intervention described in the literature to date by recruiting infants who were followed up into early adulthood [31]. In the intervention group, the authors observed a significant reduction in the prevalence of MetS in individuals aged 15–20 years, demonstrating that repeated infancy-onset dietary counseling was effective in preventing MetS in adolescence. Finally, several authors have reported that children and adolescents with MetS have an increased risk of developing type-2 diabetes and atherosclerosis in adulthood [64]. There is thus a need for long-term intervention trials specifically assessing the effects in adulthood of reducing cardiometabolic risk factor exposure during childhood. Future follow-up of the participants in the trials included in this review may reveal whether the intervention effect persists and is reflected in cardiometabolic morbidity in middle age.

Theoretically, the potential beneficial effect of nutritional education interventions on MetS can be explained by changes in the distribution of or inter-relations between individual components of MetS. This phenomenon, which has been described by other authors [65,66], should be considered when interpreting final outcomes, as intervention effects on individual components may be less pronounced than effects on clusters. Given the heterogeneity of the studies included in this review in terms of design (with/without family involvement, age of participants at recruitment), follow-up period (which ranged from 12 weeks to 20 years) and intervention type (school-based workshops, home visits to mothers, dietary counseling or dietary intervention), the aforementioned phenomenon could not be assessed. Further, more homogeneous studies should be conducted in order to clarify the influence of these types of interventions on MetS and clustered MetS components. Moreover, by identifying the most effective components of these interventions, intervention strategies could be tailored to the profiles of the individuals likely to benefit most. Stratification by age could help identify interventions better suited to children than adolescents, or vice versa. Additional research will be required to determine whether the benefits of nutritional education interventions in individuals with abdominal obesity persist into adulthood, for example by delaying the development or progression of cardiovascular disease.

## 5. Conclusions

The results of this systematic review support the beneficial effects of nutritional education interventions on abdominal obesity in children and adolescents. However, evidence regarding the impact on dyslipidemia, hypertension, fasting hyperglycemia and insulin resistance is inconclusive. 

## Figures and Tables

**Table 1:** PICOS (Population, Intervention, Comparison, Outcome and Settings) criteria [26] for the inclusion of studies

Parameter	Inclusion Criteria
Population	Children (0–10 years) and adolescents (11–21 years)
Intervention	Nutritional education interventions
Comparison	Non-exposed control group
Outcome	Metabolic risk factors
Settings	Controlled trials

**Table 2:** Effects of nutritional education interventions on abdominal obesity in 3406 children and adolescents in controlled trials.

Reference	*n*	Age ^1^	Intervention		Trial Type (Duration of Intervention)	Outcome Measure	Results at the End of the Intervention ^2^	Conclusions
Kesztyüs et al. (2017) [28]	1733 (881 F) IG 955	7.08 ± 0.63 y	**School and home.** Integration into regular curriculum and family reading material		RCT (1 y)	Incidence of abdominal obesity	Abdominal obesity (OR): IG, 0.48 (0.25,0.94)	Significantly lower OR for incidence of abdominal obesity
Gatto et al. (2017) [29]	319 (166 F) IG 172	9.03 ± 0.9 y	**Extracurricular.** Nutrition, cooking and gardening (90 min/w)		RCT (12 w)	Change in waist circumference (%)	Waist circumference: IG, −1.7%; CG, 0.1%	Significantly greater reduction in waist circumference (*p* < 0.001)
Davis et al. (2011) [33]	104 (50 F) IG 34	9.8 ± 0.7 y	**Extracurricular.** Nutrition, cooking and gardening (90 min/w) + 3 60-min family sessions		CT (12 w)	Mean waist circumference (cm)	Waist circumference: IG, 74.9 ± 13.6; CG, 77.3 ± 13.9	No significant differences
Wadolowska et al. (2019) [27]	464 (248 F) IG 319	11.9 (11.9–12.0) y	**School.** 15 h talks and workshops		CT (9 mo)	Mean difference of the change in z-WHtR and z-WC	z-WHtR: IG, −0.08 (−0.15,−0.01); CG, 0.10 (0.04,0.16) z-WC: IG, −0.05 (−0.12, −0.02); CG, 0.08 (0.03,0.19)	Significant decrease in z-WHtR (*p* < 0.001) and z-WC (*p* < 0.05)
Kong et al. (2013) [32]	51 (30 F) IG 28	IG, 15.0 ± 1 y CG, 14.06 ± 0.7 y	**School and home.** 8 sessions + DVD + print material		RCT (8 mo)	Mean change in waist circumference (cm)	Waist circumference: IG, 0.0 (−1.4,1.4); CG, 1.7 (0.4,2.9)	Significantly lower increase in waist circumference (*p* = 0.04)
Singhal et al. (2010) [34]	201 (80 F) IG 99	IG, 16.04 ± 0.41 y CG, 16 ± 0.5 y	**School.** Lectures and workshops (30 min/w) + 1 parent-teacher meeting		RCT (6 mo)	Mean change in waist circumference (cm) and waist-to-height ratio	Waist circumference: IG, −0.65 ± 3.99; CG, 0.65 ± 4.5 Waist-to-height ratio: IG, −0.005 ± 0.02; CG, 0.001 ± 0.02	Significant decrease in waist circumference (*p* = 0.02)
Nupponen et al. (2015) [31]	534 (260 F) IG 254	15–20 y	**Home.** Twice/y individualized dietary counseling		RCT (15–20 y)	RR of high waist circumference	RR of high waist circumference: IG, 0.78 (0.59–1.03)	No significant differences

Abbreviations: CG, control group; CT, controlled trial; DVD, digital versatile disc; F, female; IG, intervention group; mo, months; OR, odds ratio; RCT, randomized controlled trial; RR, relative risk; w, weeks; y, years; z-WC, waist circumference z-score; z-WHtR, waist-to-height ratio z-score. ^1^ Values represent the range or the mean ± SD, as reported in the corresponding article. ^2^ Values represent the mean and mean changes (95%CI), odds ratio ± (95%CI), relative risk (95%CI) or mean ± SD, as reported in the corresponding article.

**Table 3:** Effects of nutritional education interventions on plasma lipids in 1105 children and adolescents in controlled trials.

Reference	*n*	Age ^1^	Intervention	Trial Type (Duration of Intervention)	Outcome Measure	Results at the End of the Intervention ^2^	Conclusions
Gatto et al. (2017) [29]	319 (166 F) IG 172	9.03 ± 0.9 y	**Extracurricular.** Nutrition, cooking and gardening (90 min/w)	RCT (12 w)	Change in plasma lipids (%)	HDL: IG, 3.8%; CG, 2.3% LDL: IG, 1.5%; CG, −0.4% TG: IG, 6.3%; CG, 2.9%	No significant differences
Kong et al. (2013) [32]	51 (30 F) IG 28	IG, 15.0 ± 1 y CG, 14.06 ± 0.7 y	**School and home.** 8 sessions + DVD + print material	RCT (8 mo)	Mean change in plasma lipids (mmol/L)	HDL: IG, 0.0 (−0.09,0.09); CG, −0.04 (−0.09,0.02) TG: IG, 0.1 (−0.3,0.4); CG, 0.1 (−0.1,0.2)	No significant differences
Singhal et al. (2010) [34]	201 (80 F) IG 99	IG, 16.04 ± 0.41 y CG, 16 ± 0.5 y	**School.** Lectures and workshops (30 min/w) + 1 parent-teacher meeting	RCT (6 mo)	Mean change in plasma lipids (mg/dL)	HDL: IG, 0.31 ± 6.59; CG, −1.08 ± 6.7 TG: IG, −1.27 ± 30.2; CG, −40.4 ± 45.77	Significantly lower decrease in TG (<0.01)
Nupponen et al. (2015) [31]	534 (260 F) IG 254	15–20 y	**Home.** Twice/y individualized dietary counseling	RCT (15–20 y)	RR of high triglycerides and low HDL	RR high triglycerides: BIG, 0.71 (0.54–0.94); GIG, 1.25 (0.95–1.65) RR low HDL: 0.96 (0.75–1.23)	Significant decrease in RR of high triglycerides in boys

Abbreviations: BIG, boys intervention group; CG, control group; DVD, digital versatile disc; F, female; GIG, girls intervention group; HDL, high density lipoprotein cholesterol; IG, intervention group; LDL, low-density lipoprotein cholesterol; mo, months; RCT, randomized controlled trial; RR, relative risk; TG, triglyceride; w, weeks; y, years. ^1^ Values represent the mean or the mean ± SD, as reported in the corresponding article. ^2^ Values represent the mean, mean and mean changes (95%CI), or mean ± SD, as reported in the corresponding article.

**Table 4:** Effects of nutritional education interventions on blood pressure in 957 children and adolescents in controlled trials.

Reference	*n*	Age ^1^	Intervention	Trial Type (Duration of Intervention)	Outcome Measure	Results at the End of the Intervention ^2^	Conclusions
Gatto et al. (2017) [29]	319 (166 F) IG 172	9.03 ± 0.9 y	**Extracurricular.** Nutrition, cooking and gardening (90 min/w)	RCT (12 w)	Change in blood pressure (%)	SBP: IG, −0.6%; CG, −0.3% DBP: IG, −1.1%; CG, −3.8%	No significant differences
Davis et al. (2011) [33]	104 (50 F) IG 34	9.8 ± 0.7 y	**Extracurricular.** Nutrition, cooking and gardening (90 min/w) + 3 × 60-min family sessions	CT (12 w)	Mean blood pressure (mmHg)	SBP: IG, 101.9 ± 10.4; CG, 104.5 ± 9.8 DBP: IG, 56.5 ± 5.6; CG, 58.7 ± 6.2	Significantly lower DBP (*p* = 0.04)
Nupponen et al. (2015) [31]	534 (260 F) IG 254	15–20 y	**Home.** Twice/y individualized dietary counseling	RCT (15–20 y)	RR of high blood pressure	RR of high blood pressure: IG, 0.83 (0.7–0.99)	Significant decrease in RR of high blood pressure

Abbreviations: CG, control group; DBP, diastolic blood pressure; F, female; IG, intervention group; RCT, randomized controlled trial; RR, relative risk; SBP, systolic blood pressure; w, weeks; y, years. ^1^ Values represent the range or the mean ± SD, as reported in the corresponding article. ^2^ Values represent the mean or relative risk (95%CI), as reported in the corresponding article.

**Table 5:** Effects of nutritional education interventions on fasting glucose in 1447 children and adolescents in controlled trials.

Reference	*n*	Age ^1^	Intervention	Trial Type (Duration of Intervention)	Outcome Measure	Results at the End of the Intervention ^2^	Conclusions
Costa et al. (2017) [30]	305 (132 F) IG 126	8 y	**Home.** 9 visits to mothers during the first year of life	RCT (1 y)	Mean fasting blood glucose (mmol/L)	Fasting glucose: BIG, 4.46 ± 0.42; BCG, 4.46 ± 0.42. GIG, 4.22 ± 0.42; GCG, 4.24 ± 0.38	No significant differences
Gatto et al. (2017) [29]	319 (166 F) IG 172	9.03 ± 0.9 y	**Extracurricular.** Nutrition, cooking and gardening (90min/w)	RCT (12 w)	Change in fasting blood glucose (%)	Fasting glucose: IG, 2.2 %; CG, 1.5 %	No significant differences
Kong et al. (2013) [32]	51 (30 F) IG 28	IG, 15.0 ± 1 y CG, 14.06 ± 0.7 y	**School and home.** 8 sessions + DVD + print material	RCT (8 mo)	Mean change in fasting blood glucose (mmol/L)	Fasting glucose: IG, 0.3 (0.1,0.4); CG, 0.1 (−0.1,0.29)	Significant increase in fasting glucose (*p* = 0.04)
Davis et al. (2009) [35]	37 (17 F) IG 21	15.5 ± 1.0 y	**Extracurricular.** Nutrition, cooking and gardening (90 min/w) + 3 60-min family sessions	RCT (16 w)	Mean fasting blood glucose (mg/dL)	Fasting glucose: IG, 91.4 ± 6.55; CG, 88.7 ± 8.0	No significant differences
Singhal et al. (2010) [34]	201 (80 F) IG 99	IG, 16.04 ± 0.41 y CG, 16 ± 0.5 y	**School.** Lectures and workshops (30 min/w) + 1 parent-teacher meeting	RCT (6 mo)	Mean change in fasting blood glucose (mg/dL)	Fasting glucose: IG, −4.53 ± 7.03; CG, −2.08 ± 6.23	Significant decrease in fasting blood glucose (*p* = 0.05)
Nupponen et al. (2015) [31]	534 (260 F) IG 254	15–20 y	**Home.** Twice/y individualized dietary counseling	RCT (15–20 y)	Risk of high fasting glucose (RR)	RR high fasting glucose: IG, 0.86 (0.71–1.05)	No significant differences

Abbreviations: BCG, boys control group; BIG, boys intervention group; CG, control group; DVD, digital versatile disc; F, female; GIG, girls intervention group; GCG, girls control group; IG, intervention group; mo, months; RCT, randomized controlled trial; RR, relative risk; w, weeks; y, years. ^1^ Values represent the mean or the mean ± SD, as reported in the corresponding article. ^2^ Values represent the mean and mean changes (95%CI), mean ± SD or relative risk (95% CI), as reported in the corresponding article.

**Table 6:** Effects of nutritional education interventions on insulin resistance in 877 children and adolescents in controlled trials.

Reference	*n*	Age ^1^	Intervention	Trial Type (Duration of Intervention)	Outcome Measure	Results at the End of the Intervention ^2^	Conclusions
Costa et al. (2017) [30]	303 (131F) IG 125	8 y	**Home.** 9 visits to mothers during the first year of life	RCT (1 y)	Mean HOMA-IR	HOMA-IR: BIG, 1.15 ± 0.87; BCG, 0.92 ± 0.62 GIG, 1.06 ± 0.62; GCG, 1.41 ± 1.4	No significant differences
Kaitosaari et al. (2006) [36]	167 (85 F) IG 78	9.0 y	**Home.** Twice/y individualized dietary counseling	RCT (9 y)	Mean HOMA-IR (log)	HOMA-IR: BIG, 0.82 ± 0.29; BCG, 1.03 ± 0.41 GIG, 1.08 ± 0.45; GCG, 1.15 ± 0.44	Significantly lower HOMA-IR (*p* = 0.02)
Gatto et al. (2017) [29]	319 (166 F) IG 172	9.03 ± 0.9 y	**Extracurricular.** Nutrition, cooking and gardening (90 min/w)	RCT (12 w)	Mean change in HOMA-IR (%)	HOMA-IR: IG, 3.9%; CG, 3.8%	No significant differences
Kong et al. (2013) [32]	51 (30 F) IG 28	IG, 15.0 ± 1 y CG, 14.06 ± 0.7 y	**School and home.** 8 sessions + DVD + print material	RCT (8 mo)	Mean change in HOMA-IR	HOMA-IR: IG, 0.0 (−0.6,1.1); CG, 0.7 (−0.7,2.1)	No significant differences
Davis et al. (2009) [35]	37 (17 F) IG 21	15.5 ± 1.0 y	**Extracurricular.** Nutrition, cooking and gardening (90 min/w) + 3 × 60-min family sessions	RCT (16 w)	Mean change in HOMA-IR	HOMA-IR: IG, 5.5 ± 3.3; CG, 5.9 ± 4.8	No significant differences

Abbreviations: BCG, boys control group; BIG, boys intervention group; CG, control group; DVD, digital versatile disc; F, female; GIG, girls intervention group; GCG, girls control group; HOMA-IR, homeostasis model assessment insulin resistance; IG, intervention group; RCT, randomized controlled trial; w, weeks; y, years. ^1^ Values represent the mean or the mean ± SD, as reported in the corresponding article. ^2^ Values represent the mean, mean and mean changes (95%CI), or mean ± SD, as reported in the corresponding article.

## Supplementary Materials

The following are available online at https://www.mdpi.com/2072-6643/12/1/31/s1, Figure S1. Risk-of-bias summary: review of the authors’ judgments on each risk-of-bias item for each study; Figure S2. Risk-of-bias graph: review of the authors’ judgments on each risk-of-bias item, presented as percentages across studies.
