# Supplementary material for: Effects of Nutritional Education Interventions on Metabolic Risk in Children and Adolescents: A Systematic Review of Controlled Trials
Source: Nutrients. 2019 Dec 21;12(1):31. doi: 10.3390/nu12010031 (PMC7019568; doi:10.3390/nu12010031)
Supplement: Supplementary file 1 [file nutrients-12-00031-s001.pdf]

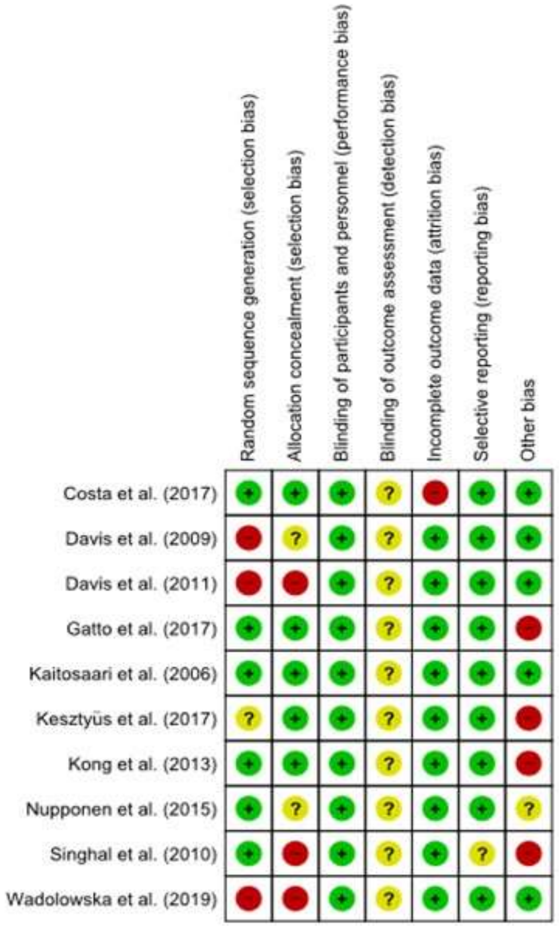

Supplemental Figure S1. Risk of bias summary: review of author's judgements on each risk of bias item for each included study.

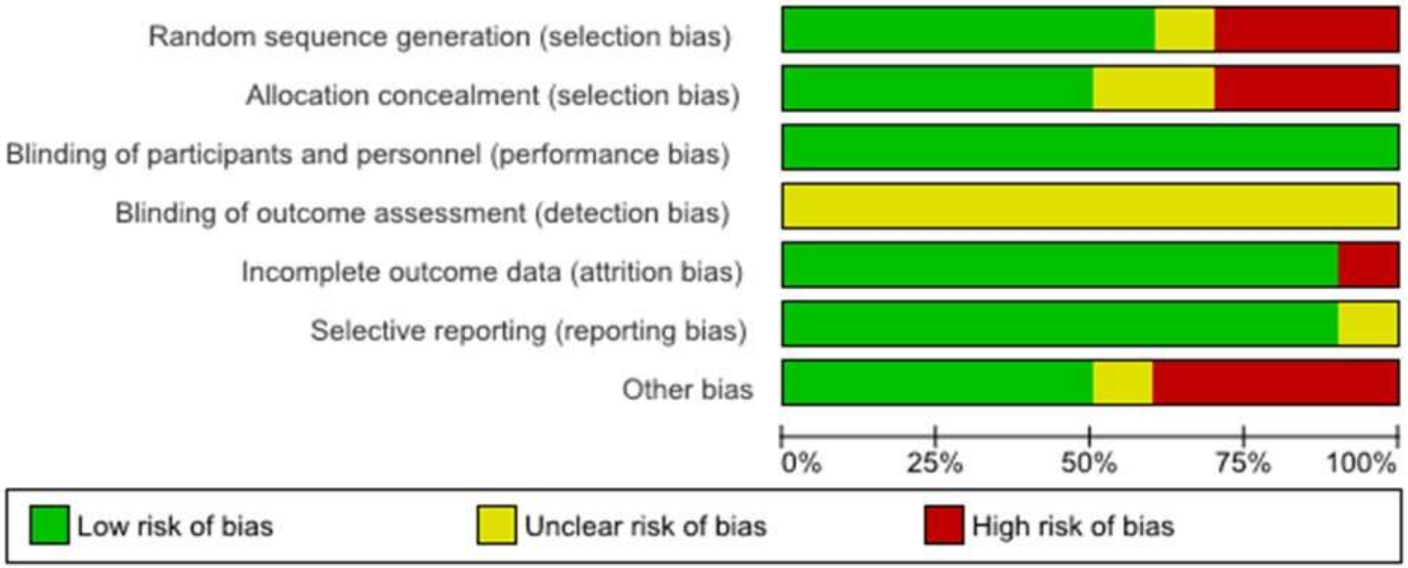

Supplemental Figure S2. Risk of bias graph: review of author's judgements on each risk of bias item presented as percentages across the studies.
